# Supplementary material for: Nutrition and Flavor Evaluation of Amino Acids in Guangyuan Grey Chicken of Different Ages, Genders and Meat Cuts
Source: Animals (Basel). 2023 Apr 2;13(7):1235. doi: 10.3390/ani13071235 (PMC10093250; doi:10.3390/ani13071235)
Supplement: Supplementary file 1 [file animals-13-01235-s001.zip › animals-2282691-supplementary.pdf]

Supplementary Materials

Table S1. The composition and content of total amino acids (mg/g dry powder)

Age	90				120				150			
Gender	male		female		male		female		male		female	
Part	leg	pectoralis	leg	pectoralis	leg	pectoralis	leg	pectoralis	leg	pectoralis	leg	pectoralis
Lys	4.31±0.21bc	5.32±0.92abc	4.63±0.34abc	5.72±0.8abc	4.65±0.38abc	5.18±0.59abc	4.77±0.8abc	6.24±0.94ab	4.13±0.65bc	5.78±0.75abc	4.29±0.69bc	6.47±0.64a
Met	3.43±0.07abc	3.41±0.18abc	3.27±0.05c	3.28±0.15bc	3.51±0.06abc	3.51±0.21abc	3.46±0.06abc	3.49±0.15abc	3.65±0.05abc	3.74±0.17a	3.68±0.07ab	3.52±0.16abc
Leu	4.35±0.05ab	2.41±0.33de	4.27±0.07abcd	2.53±0.44de	4.31±0.01abc	2.65±0.28cde	4.29±0.09abc	2.27±0.4e	4.38±0.06a	2.88±0.23bcde	4.35±0.1ab	2.72±0.39cde
Ile	4.53±0.1e	5.2±0.05c	4.47±0.09e	5.26±0.04c	4.83±0.05d	5.57±0.02b	4.65±0.04cd	5.48±0.09b	5.11±0.05c	5.82±0.09a	5.07±0.09c	5.61±0.05b
Val	2.54±0.1bc	2.83±0.13abc	2.61±0.09bc	3.12±0.33ab	2.34±0.06c	3.04±0.18ab	2.57±0.1bc	3.42±0.31a	2.65±0.05bc	3.75±0.42a	2.84±0.09abc	3.86±0.21a
Thr	3.54±0.38a	4.47±0.43a	3.64±0.78a	4.37±0.4a	3.58±0.66a	4.75±0.66a	3.74±0.38a	4.46±0.5a	3.67±0.56a	4.76±0.56a	3.87±0.47a	4.73±0.32a
His	2.53±0.32a	2.58±0.31a	2.33±0.55a	2.55±0.47a	2.67±1.04a	2.84±0.31a	2.52±0.58a	2.75±0.56a	2.87±0.13a	2.78±0.62a	2.73±0.29a	2.86±0.49a
Phe	3.68±0.12bc	3.76±0.16bc	3.66±0.18bc	3.68±0.24bc	3.89±0.12ab	4.77±0.36a	3.94±0.06ab	3.61±0.01bc	3.71±0.05bc	3.29±0.17c	3.83±0.2ab	3.79±0.18ab
Ser	4.34±0.65a	5.13±1.03a	4.25±0.88a	5.24±0.78a	4.62±0.31a	5.86±0.83a	4.78±0.44a	5.57±0.71a	4.88±0.51a	6.17±0.88a	4.92±0.8a	6.27±0.53a
Gly	6.37±0.99a	7.26±0.7a	6.82±0.78a	7.58±0.82a	5.83±0.99a	7.81±0.62a	5.42±0.42a	7.69±0.91a	6.84±0.88a	7.68±0.75a	6.77±0.69a	7.81±0.67a
Arg	5.22±0.71a	6.4±1.89a	5.43±0.71a	6.36±1.89a	5.53±0.58a	6.48±0.88a	5.72±0.62a	6.31±1.74a	6.11±0.59a	6.78±0.97a	6.12±0.64a	6.57±0.51a
Tyr	3.32±0.21ab	1.64±0.86c	3.14±0.34ab	1.43±1.04c	3.45±0.69ab	1.67±0.29c	3.36±0.92ab	2.34±0.5bc	3.62±0.83ab	2.55±0.55abc	3.55±0.38a	2.98±0.47abc
Ala	4.61±0.17a	5.14±0.65a	4.47±0.62a	5.03±1.15a	5.12±0.76a	5.52±0.87a	5.03±0.81a	5.37±0.76a	5.65±0.45a	5.54±0.72a	5.48±0.82a	5.43±1.08a
Asp	0.31±0.09d	0.54±0.29abcd	0.27±0.06d	0.77±0.09a	0.47±0.02bcd	0.62±0.05abc	0.35±0.07cd	0.68±0.1ab	0.58±0.06abcd	0.76±0.21ab	0.46±0.06bcd	0.77±0.1a
Glu	17.67±0.78abc	18.43±0.42ab	16.76±0.67bc	16.56±0.88c	16.28±1.04c	18.78±0.75a	16.03±0.93c	17.62±0.71abc	17.65±1.02abc	19.16±0.45a	16.84±0.91bc	18.44±0.45ab
Pro	3.36±0.54a	2.71±0.69a	3.45±0.33a	3.66±0.51a	3.47±0.7a	2.01±0.58a	3.51±0.43a	3.87±0.99a	3.77±0.36a	4.07±1.18a	3.85±0.91a	4.02±0.92a
Cys	1.57±0.64a	1.23±1.05a	1.63±0.59a	1.34±0.69a	1.64±0.69a	1.39±0.71a	1.72±1.07a	1.23±0.33a	1.65±0.56a	1.28±0.33a	1.77±0.76a	1.14±0.17a

Different letters superscript in the same row indicates significant difference, $p < 0.05$.

Table S2. The composition and content of free amino acid (ug/g fresh meat)

Age	90				120				150			
Gender	male		female		male		female		male		female	
Part	leg	pectoralis	leg	pectoralis	leg	pectoralis	leg	pectoralis	leg	pectoralis	leg	pectoralis
Glu	260.37±72.33ab	240.95±42.32abc	331.61±64.26a	225.72±35.09abc	336.62±61.77a	297.82±74.48a	255.37±29.25a	217.53±8.56abc	223.07±22.57abc	226.5±23.6abc	170.84±26.66c	176.13±24.6bc
Asp	181.19±35.05a	91.87±6.00bcd	156.21±22.38ab	89.9±7.23bcd	155.87±27.63ab	112.16±25.36abc	169.98±11.78a	58.23±0.61d	107.63±4.94abc	84.84±7.38cd	95.39±2.42abcd	65.65±3.01cd
Asn	393.86±45.62ab	340.47±62.52ab	313.03±62.09ab	361.84±60.03ab	362.3±84.45ab	436.03±109.77a	379.17±78.4ab	289.95±18.51ab	308.04±46.37ab	392.98±60.15ab	222.31±32.39b	315.49±74.51ab
Ala	351.47±5.85ab	292.74±51.3ab	335.79±37.28ab	306.33±78.8ab	377.48±6.46a	351.97±31.01ab	371.26±48.87a	252.77±82.02ab	303.86±56.97ab	267.98±96.92ab	234.8±69.45ab	195.74±63.92b
Ser	230.59±35.04a	154.67±20.07abcd	224.46±36.28a	163.36±11.78abcd	235.99±59.97a	166.69±46.03abcd	192.65±46.07abc	125.26±2.64cd	212.12±33.76ab	142.71±4.4bcd	151.26±11.79abcd	110.95±10.03d
Gly	173.52±46.47a	68.96±0.78de	143.94±28.56abc	85.33±7.03cde	140.96±35.73abc	96.21±18.07abcde	156.77±33.63abc	65.93±1.92de	170.72±25.07ab	87.55±13.34bcde	105.78±7.8abcd	50.09±2.56e
Pro	78.96±14.17ab	46.89±0.33bcd	64.4±1.34abcd	44.22±1.69cde	99.09±19.52a	73.96±12.65abcd	85.17±3.89ab	42.85±3.12de	76.65±4.18abc	47.98±1.01abcde	37.22±0.84e	35.82±1.65e
Thr	90.66±10.81ab	86.44±5.78abc	87.44±7.95abc	78.04±7.33bcd	128.26±1.94a	120.28±17.21a	96.79±2.11ab	82.73±0.55bcd	82.36±3.05bcd	86.37±1.53abc	43.31±0.12d	58.45±5.31cd
Gln	948.95±244.82ab	246.56±10.43cd	916.06±79.97ab	244.67±3.74c	1087.14±269.23a	314.08±44.38bcd	738.23±72.69abc	191.06±32.46d	1032.01±96.18a	258.14±9.35bcd	593.04±20.27abcd	187.11±2.59d
Arg	133.13±21.67ab	134.89±25.66ab	117.98±25.45abcd	96.14±4.54bcd	184.39±53.68a	110.58±12.34abcd	175.66±44.23a	151.08±19.3a	129.13±21.28abc	98.51±20.23bcd	87.49±1.42cd	51.14±6.15d
His	375.76±63.07	343.48±42.7a	185.84±34.7	208±37.11	527.79±1	316.4±10.25	157.92±21.	237.03±15.2	363.01±27.	466.55±104	160.25±3.48	147.28±1.

	ab	bc	6cde	bcde	42.17a	abcd	03de	4abcde	3ab	.08a	de	31e
Met	42.76±0.94cd	79.57±3.11ab	41.72±3.9cd	81.58±2.36ab	51.51±1.46bcd	84.86±7.87a	55.93±6.55abcd	56.75±9.24abc	36.46±6.24cd	75.98±3.88ab	20.44±1.49d	60.83±6.05abc
Val	70.94±6.07cdef	97.38±5.63abc	63.12±0.92def	99.09±0.84ab	87.56±8.99abcd	118.58±18.3a	84.09±0.89abcde	77.22±6.05bcdef	47.37±6.93ef	91.23±1.29abc	29.39±0.24f	74.16±3.83cdef
Ile	59.45±2.6cde	90±4.45ab	53.75±1.26de	89.68±1.4ab	70.34±2.99abcd	111.39±12.18a	68.12±4.54abcde	64.94±8.83bcde	38.69±3.74de	86±2.46abc	24.21±3.04e	65.97±4.74bcde
Phe	58.16±6.29de	96.31±4.29ab	62.6±3.58cde	111.76±6.71a	71.47±8.25abcde	106.13±16.61a	64.9±0.23bcde	72.88±6.76abcd	48.35±6.76de	90.16±3.36abc	29.1±0.9e	69.57±3.57abcd
Trp	18.29±2.84cd	28.32±0.39a	19.6±3.31bcd	26.05±2.81ab	20.48±1.73bcd	26.83±2.02ab	20.52±3.51bcd	20.74±5.65abcd	15.42±2.58d	25.76±0.48abc	8.39±0.03d	21.36±1.58abcd
Leu	94.81±5.45def	174.93±9.55ab	99.38±4.12cdef	183.25±9.52a	119.8±9.07abcdef	191.53±28.67a	109.6±7.05bcdef	126.65±11.1abcde	75.95±8.58ef	161.87±2.53abc	40.82±0.88f	136.66±5.4abcd
Lys	345.77±65.77a	269.45±27.5ab	243.31±2.7abc	267.58±31.05ab	330±41.37a	380.4±96.09a	328.11±14.69a	222.78±9.77bc	193.16±9.71bc	266.9±25.66ab	85.35±3.54c	185.44±16.22bc
Tyr	71.96±3.66cde	116.69±8.82abc	60.23±5.1de	117.58±6.17ab	76.13±9.26bcde	129.67±16.59a	75.81±5.03bcde	77.02±7.37abcde	46.94±2.65e	112.81±10.53abc	24.14±1.11e	87.33±1.24abcd
GABA	0.77±0.22abcd	0.97±0.33ab	0.87±0.17abc	1.09±0.33ab	0.78±0.36abcd	1.42±0.42a	0.67±0.18bcd	0.63±0.01bcd	0.52±0.05d	1.00±0.15ab	0.53±0.08cd	1.02±0.2cd
Orn	2.27±0.31b	0.58±0.27d	1.86±0.31bcd	1.11±0.73bcd	3.88±0.35a	0.72±0.27cd	5.05±0.81a	0.95±0.43cd	4.00±0.29a	1.2±0.29bcd	4.62±0.26a	1.98±0.53bc

Different letters superscript in the same row indicates significant difference, $p < 0.05$.

Table S3. The variance analysis of three factors (age, gender, meat cut) of total amino acids

P value	P(Age)	P(Gender)	P(Meat cut)	P(Age*Gender)	P(Age*Meat cut)	P(Gender*Meat cut)	P(Age*Gender*Meat cut)
Lys	0.72	0.05	0.00	0.91	0.20	0.26	0.74
Met	0.00	0.04	0.85	0.58	0.89	0.47	0.32
Leu	0.11	0.29	0.00	0.58	0.33	0.58	0.42
Ile	0.00	0.00	0.00	0.03	0.02	0.76	0.02
Val	0.00	0.01	0.00	0.63	0.00	0.49	0.65
Thr	0.51	0.97	0.00	0.94	0.94	0.41	0.95
His	0.35	0.62	0.50	0.97	0.91	0.67	0.98
Phe	0.00	0.11	0.60	0.00	0.01	0.02	0.00
Ser	0.04	0.98	0.00	0.97	0.76	0.90	0.85
Gly	0.21	0.85	0.00	0.60	0.10	0.82	0.94
Arg	0.48	1.00	0.04	0.98	0.86	0.71	1.00
Tyr	0.02	0.67	0.00	0.63	0.26	0.35	0.75
Ala	0.10	0.63	0.30	1.00	0.61	0.99	1.00
Asp	0.01	0.94	0.00	0.30	0.39	0.03	0.78
Glu	0.03	0.00	0.00	0.50	0.03	0.27	0.65
Pro	0.05	0.05	0.47	0.30	0.42	0.09	0.28
Cys	0.98	0.96	0.10	0.97	0.94	0.74	0.95

Table S4. The variance analysis of three factors(age, gender, meat cut) of free amino acids

P value	P(Age)	P(Gender)	P(Meat cut)	P(Age*Gender)	P(Age*Meat cut)	P(Gender*Meat cut)	P(Age*Gender*Meat cut)
Glu	0.00	0.03	0.05	0.02	0.21	0.37	0.41
Asp	0.00	0.01	0.00	0.90	0.00	0.14	0.01
Asn	0.11	0.01	0.24	0.62	0.15	0.69	0.06
Ala	0.00	0.05	0.02	0.34	0.75	0.58	0.44
Ser	0.02	0.01	0.00	0.15	0.84	0.48	0.87
Gly	0.30	0.01	0.00	0.05	0.74	0.57	0.06
Pro	0.00	0.00	0.00	0.04	0.03	0.19	0.01
Thr	0.00	0.00	0.26	0.00	0.00	0.99	0.27
Gln	0.26	0.00	0.00	0.03	0.57	0.01	0.22
Arg	0.26	0.00	0.00	0.03	0.57	0.01	0.22
His	0.39	0.00	0.66	0.14	0.09	0.06	0.00
Met	0.00	0.00	0.00	0.00	0.00	0.01	0.00
Val	0.00	0.00	0.00	0.01	0.00	0.06	0.00
Ile	0.00	0.00	0.00	0.00	0.00	0.00	0.00
Phe	0.00	0.00	0.00	0.00	0.00	0.23	0.01
Trp	0.00	0.00	0.00	0.08	0.00	0.20	0.15
Leu	0.00	0.00	0.00	0.08	0.00	0.20	0.15
Lys	0.00	0.00	0.40	0.42	0.00	0.72	0.00
Tyr	0.00	0.00	0.00	0.01	0.00	0.01	0.00
GABA	0.28	0.19	0.00	0.02	0.41	0.19	0.16
Orn	0.00	0.00	0.00	0.14	0.00	0.85	0.05
